# SARS-CoV-2 infection status in corneal preservation solution and COVID-19 prevalence after corneal transplantation

**DOI:** 10.1038/s41598-024-53863-x

**Published:** 2024-02-14

**Authors:** Yuki Wasai, Naoyuki Yamada, Nobuaki Ariyoshi, Aiko Haraguchi, Masahiko Funatsu, Masanori Mikuni, Riku Nakamura, Ayano Sakuma, Fumiaki Higashijima, Nanako Iwamoto, Shinichiro Teranishi, Mitsuaki Nishioka, Takahiro Yamasaki, Kazuhiro Kimura

**Affiliations:** 1 Department of Ophthalmology, Yamaguchi University Graduate School of Medicine, 1-1-1 MinamiKogushi, Ube, Yamaguchi 755-8505 Japan; 2 Department of Ophthalmology, Kyushu University Hospital, 3-1-1 Maidashi, Higashi-ku, Fukuoka, 812-8582 Japan; 3 Division of Laboratory, Yamaguchi University Hospital, 1-1-1 MinamiKogushi, Ube, Yamaguchi 755-8505 Japan; 4 Department of Oncology and Laboratory Medicine, Yamaguchi University Graduate School of Medicine, 1-1-1 MinamiKogushi, Ube, Yamaguchi 755-8505 Japan

**Keywords:** Corneal diseases, Vision disorders

## Abstract

The potential risks associated with organs from COVID-19-infected donors were unclear. To determine the SARS-CoV-2 infection status of corneas transplanted during the COVID-19 pandemic, we performed a polymerase chain reaction (PCR) using the corneal preservation solution that was used for corneal transplantation. We also examined the postoperative health status of the recipients. This study included 144 transplants in 143 eyes. Ninety-nine eyes of imported corneas and 10 of the 14 corneas donated in the prefecture were PCR tested at our hospital, and all were SARS-CoV-2 negative. All corneal transplants were performed after confirming their SARS-CoV-2 negativity by a PCR using a corneal preservation solution at our hospital or a nasopharyngeal swab at a previous facility. Despite postoperative steroid administration, no patient developed COVID-19 infection until discharge. Hence, if the donor's nasopharyngeal swab test is SARS-CoV-2 negative, COVID-19 infection in the recipient due to corneal transplantation may be prevented. Since corneal transplant recipients are susceptible to infection due to prolonged steroid administration and are at high risk for severe diseases if infection occurs, SARS-CoV-2 detection testing using nasopharyngeal swabs in donors should be performed.

## Introduction

Cornea is an avascular, colorless, transparent tissue situated on the surface of the eye. It refracts the light entering the eye, focusing it on the retina. However, some factors, such as trauma or infection can cause permanent edema or clouding of the cornea, leading to the loss of transparency and abnormal corneal shape, which gradually results in decreased visual function. In some cases, corneal transplantation can be performed to restore visual function by replacing the irreversibly cloudy or deformed host cornea with a graft.

Corneal transplants are the most frequently performed transplant surgeries in the human body. Over 49,000 transplants have been carried out in the United States^1^. Similarly, in Japan, approximately 2,600 corneal transplants are performed each year^2^. In contrast, approximately 1,700 kidney transplants^3^, 400 liver transplants^4^, 50 heart transplants^5^, and 70 lung transplants^6^ are performed yearly in Japan. Thus, the number of corneal transplants performed surpasses other organ transplants.

While corneal transplants have recently become safer, various intraoperative or postoperative complications can occur. Postoperatively, graft rejection, graft failure, infection, and recurrence of the underlying disease are major problems. As with other organ transplants, graft rejection is a serious complication. Therefore, postoperative immunosuppressive therapy is administered to reduce the chances of rejection of the transplanted cornea. Recent advances in immunosuppressive agents have reduced the incidence of rejection in corneal transplantation; nevertheless, they must be used continuously for a certain period of time. As a results, the transplanted cornea is infected by pathogenic microorganisms, such as bacteria, fungus, or virus, due to immunosuppression, and sometimes the infection is severe^7,8^. In addition to opportunistic infections, other infection routes specific to organ transplantation, such as initial infection or reinfection via the donor, must also be considered.

Severe acute respiratory syndrome coronavirus 2 (SARS-CoV-2) is a novel coronavirus. COVID-19 is a severe acute respiratory syndrome infection caused by SARS-CoV-2 that was first reported in Wuhan, China in December 2019, following which, it rapidly spread worldwide. The main symptoms of COVID-19 include fever, cough, fatigue, myalgia, and dyspnea, with some reports of headache, conjunctivitis, and diarrhea^9–11^. Recent studies have reported that immunosuppressed individuals infected with SARS-CoV-2 are particularly susceptible to the severe form of COVID-19^12^. The impact of the COVID-19 epidemic on various organ transplants was significant worldwide because the potential risks associated with organs from COVID-19-infected donors were unclear. In the United States, kidney transplantation was the most severely affected, with a reported decrease in the number of kidney, lung, heart, and liver transplants from deceased donors^13–16^. In Japan, the Ministry of Health, Labor, and Welfare issued a notice in April 2020 stating that potential donors with a SARS-CoV-2 positive polymerase chain reaction (PCR) test result should not be used for organ transplants, leading to an overall decrease in the number of organ transplants. This decline was also seen in corneal transplants, with the number of corneal donors plummeting from 720 before the COVID-19 pandemic to 505 after the pandemic between FY 2018 and FY 2021, respectively. Similarly, the number of transplanted eyes also decreased from 1155 before the COVID-19 pandemic to 814 eyes after the pandemic^17^. However, of note, corneal transplantation procedures have now resumed at many institutions. Although corneal transplant procedures were back to normal levels, there was a shortage of suitable corneal tissue. The discontinuation of post-mortem testing of COVID-19 in the U.S. has made it possible to obtain many imported corneas^18^.

The primary route of coronavirus infection is respiratory droplet infection; the angiotensin-converting enzyme 2 (ACE-2) receptor, which has been identified as the entry receptor for SARS-CoV-2, is found not only in the airway mucosa but also in the conjunctival cell membrane^19^. There are several reports suggesting that the conjunctiva may harbor SARS-CoV-2 and be directly or indirectly involved in viral infection^19^. SARS-CoV-2 infection could be transmitted via the donor corneal tissue. Currently, there are no clear guidelines for donor recovery after and testing for SARS-CoV-2 infection. Therefore, to investigate the status of SARS-CoV-2 infection in transplanted corneas under the current COVID-19 pandemic, we collected the corneal preservation fluid from donor corneas and performed Reverse-transcription PCR (RT-PCR) to determine the presence of COVID-19 infection. In addition, the postoperative health status of the recipients was also assessed.

## Results

We examined if the presence of SARS-CoV-2 infection in corneal preservation solutions was detectable by PCR. The General data was shown in Table 1. This study included 144 corneal transplants in 143 eyes. Of these, 130 transplants were performed in 129 eyes using imported corneas and 14 transplants were performed in 14 eyes using eyes donated within the prefecture. Among a total of 144 procedures, the corneal transplantation procedures included 89 PKPs, 39 Descemet’s strippings automated endothelial keratoplasties (DSAEKs), 5 deep anterior lamellar keratoplasties (DALKs), 4 sclerokeratoplasties (SPKs), 5 laminar keratoplasties (LKPs), 1 ring transplant, and 1 corneal epithelioplasty. The male:female ratio was 69:75, the mean age (± standard deviation) was 70 ± 17 years, and the right: left eye ratio was 71:73. One eye in which an imported corneal PCR was performed was used to perform a ring corneal transplant and penetrating keratoplasty (PKP). Finally, of the 129 imported corneas, 30 eyes were confirmed negative by PCR testing using nasopharyngeal swab fluid in the United States. The remaining 99 imported corneas were subjected to corneal preservation solution PCR for detection of SARS-CoV-2 using the Ampdirect™ 2019-nCoV detection kit (Shimadzu Corporation, Japan) (Shimadzu method) at 7 ± 1 day after corneal collection. All 99 eyes were negative for SARS-CoV-2. Four of the 14 corneas donated in the prefecture were confirmed negative by PCR using nasopharyngeal swab fluid at the previous facility. The remaining 10 donated corneas were subjected to corneal preservation solution PCR testing using the Shimadzu method on the same day as the cornea was collected. Postoperatively, all 10 eyes tested negative for SARS-CoV-2.

**Table 1:** General data.

Subject	143 eyes, 144 corneal transplants
Sex (male:female)	69 (47.92%):75 (52.08%)
Age (mean ± SD, years)	70 ± 17
Eye (right:left)	71 (49.31%):73 (50.69%)
Period	From November 2020 to October 2022

*SD* standard deviation.

The presence of preoperative COVID-19 infection in the recipients was confirmed before admission in all cases. All recipients were admitted after PCR testing was performed using nasopharyngeal swabs by the Shimadzu method described above and confirmed negative for SARS-CoV-2. Regarding preoperative subjective symptoms that were suspicious for coronas, fever, cough, malaise, myalgia, and dyspnea were not observed in all patients.

The mean temperature and SpO2 at admission were 36.40 ± 0.42 °C and 97.39 ± 0.93%, respectively, and at discharge, the mean temperature and SpO2 were 36.31 ± 0.42 °C and 97.39 ± 1.05% at room temperature, respectively. Here, at discharge refers to the date of last measurement. The mean postoperative days for which measurements were taken were 7.60 ± 4.02 days for temperature and 9.43 ± 3.77 days for SpO2.

The mean postoperative hospital stay was 10.26 ± 3.81 days. After corneal sampling, corneal transplantation took an average of 13 ± 30 days. There were no intraoperative or postoperative corneal transplantation related complications. At discharge, no patient had any COVID-19 infection suspicious symptom, such as fever, cough, fatigue, myalgia, or dyspnea.

## Discussion

Prevention of postoperative infection is crucial after corneal transplantation because of the patient’s susceptibility to infection caused by prolonged local and systemic steroid administration. In the present study, we performed PCR tests to determine the SARS-CoV-2 infection status of corneas transplanted during the corona pandemic using corneal preservation solution from some of the preoperative imported corneas and some from the corneas donated within the prefecture. We confirmed that all patients were negative for SARS-CoV-2. Subsequently, corneal transplantation was performed, and despite postoperative local and systemic steroid administration, no COVID-19 infection was detected in any patient until discharge. In other words, we could prevent postoperative COVID-19 infection by performing PCR testing of corneal preservation solution and confirming that the test was negative. For the 30 imported corneas and 4 donor eyes within the prefecture, COVID-19 negativity of the donors was confirmed using nasopharyngeal swab fluid; hence, PCR of the corneal preservation solution was not performed for them. On the other hand, for the 99 imported corneas and 10 in-prefecture donated corneas, the presence or absence of donor COVID-19 infection was not confirmed prior to corneal transplantation, and PCR was performed for them to confirm negative results. Both patients did not develop COVID-19 infection or disease after transplantation. Donor nasopharyngeal swab testing may also prevent COVID-19 infection from corneal transplantation.

The Centers for Disease Control and Prevention (CDC) lists immunocompromised patients, including those who require immunosuppressive therapy after organ transplantation, as being at high risk for severe illness due to SARS-CoV-2 infection. However, compared with the general population, immunosuppressed patients have not been reported to have a significantly increased risk of COVID-19 infection^20^. There are no direct reports of immunosuppressive treatment increasing the risk of COVID-19 infection. Contrarily, there have been several reports of an increased risk of severe disease when immunocompromised individuals were infected with SARS-CoV-2^12^. Furthermore, in a report examining immunosuppressive therapy and its impact on the severity and outcome in patients diagnosed with SARS-CoV-2, exposure to selective immunosuppressive agents, tumor necrosis factor inhibitors, interleukin inhibitors, calcineurin inhibitors, other immunosuppressive agents, hydroxychloroquine, and chloroquine were not associated with COVID-19 severity; however, exposure to glucocorticoids has been reported to be associated with an increased risk of hospitalization and death^21–23^. The relative risk of serious outcomes has also been reported to be higher with higher cumulative exposure to glucocorticoids in the 120 days prior to a positive SARS-CoV-2 diagnosis^22^. Among immunosuppressive treatments, steroids appear to have the greatest impact on the risk of COVID-19 severity. We routinely administer local and systemic postoperative steroids to all corneal transplant recipients to prevent rejection in corneal transplant surgery. In the present study, no cases of COVID-19 infection were observed despite the high risk of severe COVID-19 infection during post-operative steroid administration after corneal transplantation. The negative preoperative PCR tests of preoperative pharyngeal swab fluid and donor corneal preservation solution were negative, suggesting that infection from the donor cornea may have been suppressed. There were also several reports of corneal graft rejection after corneal transplantation related to COVID-19 vaccine administration. Those were reported to have been treated with topical steroids^24^. Although none were observed in the present case, it is possible that they were prevented by the use of topical steroid administration in all cases to prevent postoperative rejection. Furthermore, it is important to require not only preoperative testing for SARS-CoV-2 infection but also other viral tests in corneal transplants that require immunosuppression with steroids.

The viral load of nasopharyngeal swabs and sputum peaked at 5–6 days after onset (104–107 copies per mL), and even sputum samples taken 8 days after onset from patients who died were reported to have very high viral load (1–34 × 1011 copies/mL)^25^. SARS-CoV-2 ribonucleic acid (RNA) is detectable in nasopharyngeal swab fluid from patients who died of coronavirus pneumonia in 70.3% of cases within 2 h after death and 66.6% of cases within 24 h after death^26^, and RNA was detectable in nasopharyngeal swab fluid until 128 h after death^27^. Regarding SARS-CoV-2 survival outside of the nasopharyngeal swab fluid, RT-PCR on retinal samples from deceased patients with confirmed COVID-19 revealed viral RNA of SARS-CoV-2 in 3 of 14 eyes^28^, and PCR on vitreous fluid 3 months after death detected coronal SARS-CoV-2 RNA^29^. There have been several reports of coronas being detected in conjunctival swabs from patients with coronas, and there is also a report that an eye swab from a patient with conjunctivitis due to corona infection was positive for SARS-CoV-2 by RT-PCR even about two weeks after the onset of conjunctivitis^30–33^. In the present study, PCR testing was performed 7 ± 1 days after corneal collection for the imported corneas and on the same day as corneal collection for the corneas donated within the prefecture using corneal preservation solution, and all cases were confirmed to be negative for SARS-CoV-2. After ex vivo infection of the harvested corneas with SARS-CoV-2 solution, RNA was extracted from the tissue and culture medium and quantified by RT-qPCR at 30 min and 24 h after infection, and a significant increase was observed in the central corneal area, with fluctuations in RNA copies^34^. In this study, the presence of SARS-CoV-2 in the preservation solution can be ruled out; however, the survival of SARS-CoV-2 in the cornea cannot be ruled out. Furthermore, of the 101 corneas that tested positive for SARS-CoV-2 in postmortem nasopharyngeal swabs, only 3 were positive for SARS-CoV-2 RNA in the corneal preservation solution^35^. It is possible that even if the donor had been infected with SARS-CoV-2 in the past, its RNA may not have been detected in the corneal preservation solution.

Even if the donor is already infected with SARS-CoV-2, RNA may not be detected in corneal preservation solution. An RT-PCR of nasopharyngeal swab fluid from corneal donors without symptoms revealed that 12 of 85 samples were positive for COVID-19^36^. In addition, RNA of SARS-CoV-2 was detected in 2 (0.3%) corneal specimens from 588 donors with no medical or epidemiological relevance for COVID-19, and RNA was also detected in their corneal preservative fluid^37^. These findings suggest that some asymptomatic COVID-19 infected corneal donors are present. Furthermore, there have been reports of positive SARS-CoV-2 RNA in corneal tissue and cell cultures of stored fluid; however, no cytotoxic effect or plaque formation by the virus has been reported, and the risk of infection from corneal transplantation may be low^37^. However, prevention of postoperative infection is crucial because corneal transplant recipients are susceptible to infection due to prolonged local and systemic steroid administration and are at high risk for severe disease when infection does occur. In Japan, corneal transplantation relies on imported corneas from US. Post-mortem testing for COVID-19 had been discontinued in the U.S.^18^, so it would be even more useful to use preservation solution to check for infection in such cases. In addition, we consider that using donor corneal preservation fluid to check for infection is simpler and safer than using nasopharyngeal swab fluid of the deceased patient, including the reduced risk of secondary infection of the collector. Therefore, it is important to mandate COVID-19 detection testing in donor corneal preservation solution as along with other viral tests.

This study has the following limitations. First point is that the sample size is limited, and there is no denying that this point may have biased the results. Due to the small number of cases in this study, it is possible that COVID-19 positive donors were not initially included. Second, because the donor corneas were obtained for use in corneal transplantation surgery, we did not perform PCR testing for the presence of COVID-19 infection using corneal tissue. However, since it has been reported that SARS-CoV-2 RNA was detected in corneal specimens and RNA was also detected in their corneal preservation fluid^37^, we performed PCR testing using corneal preservation fluid in this study to confirm COVID-19 negativity.

## Methods

The data was examined retrospectively based on their medical records. This study complied with the guidelines of the Declaration of Helsinki and Ethical Guidelines for Medical and Biological Research Involving Human Subjects. This study was approved by the institutional review board of the Yamaguchi University Hospital; Research Ethics Committee (REC) of Yamaguchi University Hospital (approval no. 2023-132). The REC waived the need for written informed consent for all subjects and approved the use of an opt-out consent method for this study.

**Figure 1:**
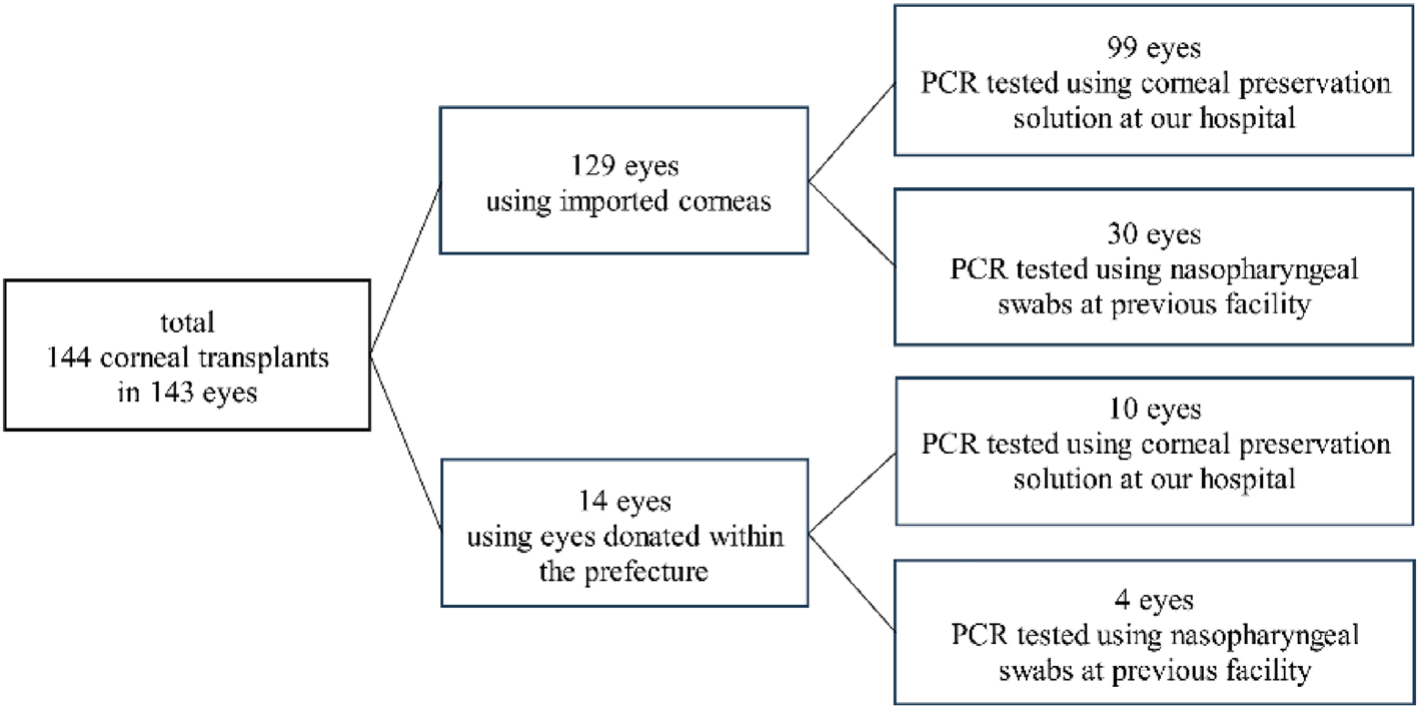
Breakdown of PCR. Of the 144 cases in 143 eyes, 130 cases were transplants in 129 eyes using imported corneas, and 14 cases were transplants in 14 eyes using eyes donated within the prefecture. Of the 130 cases of 129 imported corneas, PCR was performed on 99 eyes and 100 cases at our hospital. In 30 cases of 30 eyes, PCR was confirmed to be negative using a nasopharyngeal swab performed in the United States, so it was not performed. Of the corneas donated within the prefecture, PCR was performed on 10 of the 10 eyes at our hospital. PCR was not performed at our hospital because four of the eyes donated within the prefecture had been confirmed negative by PCR at the previous facility.

This study included 144 corneal transplants in 143 eyes performed between November 2020 and October 2022 at the Department of Ophthalmology, Yamaguchi University Hospital. One eye in which an imported corneal PCR was performed was used to perform two cases of ring corneal transplantation and PKP. Regarding the recipients' history of infection, 1 recipient had a positive for hepatitis B virus antibody (HBc Ab) test, 2 recipients were positive for hepatitis C virus antibody (HCV Ab), 7 recipients had a positive treponema pallidum hemagglutination (TPHA) test, 4 recipients had a positive rapid plasma regain (RPR) test, 29 recipients had diabetes, 62 recipients had hypertension, and no patient had human immunodeficiency virus (HIV). The recipients had the following primary eye diseases—bullous keratopathy, graft failure, vitiligo, and keratoconus. Donor corneal infections in all transplants are checked via blood tests. All the aforementioned viral tests were negative. Imported corneas were confirmed negative according to the donor screening guidelines of the Eye Bank Association of America (EBAA). The domestic donor corneas were also negative for all the aforementioned viral tests. Of the 144 transplants in 143 eyes, 130 transplants in 129 eyes were performed using imported corneas, and 14 transplants in 14 eyes were performed using eyes donated within the prefecture. Of the 130 transplants of 129 imported corneas, 100 transplants of 99 eyes underwent corneal transplantation after performing PCR using corneal preservation solution at our hospital, remain 30 transplants in 30 eyes were not subjected to PCR at our hospital because the PCR-negative results of the imported corneal donors had already been confirmed in the U.S. using nasopharyngeal swab fluid. Of the corneas donated within the prefecture, 10 were from 10 eyes for which PCR was performed at the hospital. Four cases in four eyes were not subjected to PCR as they were confirmed negative by the previous facility. (Fig. 1). In all cases of corneal transplantation surgery at our hospital, steroids are administered locally and systemically intraoperatively and postoperatively to prevent graft rejection. For systemic steroid therapy, Rinderon 4 mg intraoperatively + Rinderon 2 mg/day for 4 days postoperatively is administered. For local steroid therapy, postoperative steroid eye drops are prescribed for a prolonged period of time.

SARS-CoV-2 nucleic acid detection test (PCR test method) at our hospital is performed as followes: On a clean bench, 750 µL of corneal preservation solution is collected and the specimen is submitted to the Yamaguchi University School of Medicine Laboratory. Ocular Preservation Solution, II (EP, II) (Kaken Pharmaceutical Co., Ltd.) is the corneal preservation solution used for corneas donated within the prefecture. RT-PCR was performed using the Ampdirect™ 2019-nCoV detection kit (Shimadzu Corporation, Japan). QuantStudio®5 (Applied Biosystems, USA) and Real-time one-step RT-PCR (TaqMan probe method) was used.

(1) First, 5 μL of nasopharyngeal swab solution (or corneal preservation solution) was mixed with 5 μL of pretreatment solution and heated at 90 °C for 5 minutes.

(2) Next, 15 µL of RT-PCR reaction reagent (prepared by mixing solutions A to C) and 10 µL of the solution were mixed (1) and dispensed into a 96-well plate; subsequently, they were inserted in the real-time PCR system (42 °C, 10 min, 1 cycle → 95 °C, 1 min, 1 cycle → 95 °C, 5 sec, 45 cycles → 60 °C, 30 sec, 45 cycles).

**Figure 2:**
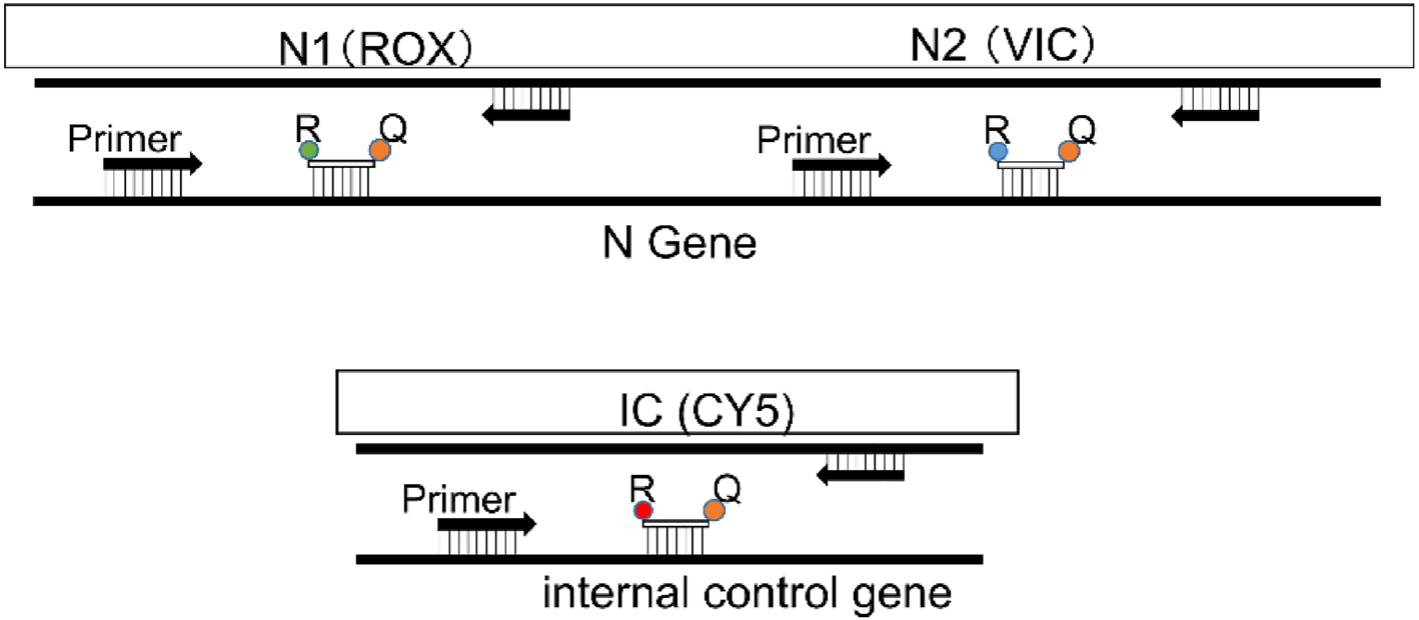
Detection part in Shimadzu method. PCR is performed at three locations simultaneously (N Gene: N1 (ROX), N2 (VIC), internal standard DNA: IC (CY5)). RT-PCR was performed by one-step RT-PCR (TaqMan probe method).

PCR was performed at three locations simultaneously (N Gene: N1 (ROX), N2 (VIC), internal standard DNA: IC (CY5)). RT-PCR was performed by one-step RT-PCR (TaqMan probe method) (Fig. 2). The detection sensitivity of the reagent was nine copies/test, which means that the virus could be detected if there were more than nine copies of the virus in 5 μL of corneal preservation solution.

## Data Availability

The datasets used and/or analysed during the current study available from the corresponding author on reasonable request.
